# 竹叶提取物中7种黄酮成分含量测定及抗氧化分析

**DOI:** 10.3724/SP.J.1123.2024.01008

**Published:** 2024-10-08

**Authors:** Liling GU, Xi YAO, Rongmiao AN, Xuefeng GUO

**Affiliations:** 国际竹藤中心, 国家林业和草原局/北京市共建竹藤科学与技术重点实验室, 北京 100102; International Centre for Bamboo and Rattan, Key Laboratory of National Forestry and Grassland Administration/Beijing for Bamboo and Rattan Science and Technology, Beijing 100102, China

**Keywords:** 高效液相色谱, 竹叶提取物, 黄酮, 抗氧化, high performance liquid chromatography (HPLC), bamboo-leaf extracts, flavonoid, antioxidant

## Abstract

为研究不同竹叶提取物中黄酮成分含量及其与抗氧化活性之间的关系,以2种市售竹叶提取物以及以毛竹等7种竹种的竹叶为材料制备的竹叶提取物共9个样品为研究对象,建立了同时测定竹叶提取物中7种黄酮成分(荭草苷、异荭草苷、牡荆苷、异牡荆苷、苜蓿素、木犀草素和木犀草苷)的高效液相色谱(HPLC)方法,采用SymmetryShield^TM^ RP8色谱柱(250 mm×4.6 mm, 5 μm),以乙腈为流动相A, 0.5%(v/v)乙酸水溶液为流动相B进行梯度洗脱。采用1,1-二苯基-2-三硝基苯肼(DPPH)和羟基自由基清除试验评价竹叶提取物的抗氧化活性,以半抑制浓度(IC_50_)为评价指标,以抗氧化剂2,6-二叔丁基对甲酚(BHT)和叔丁基对苯二酚(TBHQ)为阳性对照,并利用Person相关性分析各黄酮成分含量与抗氧化的关系。结果表明:建立的HPLC测定方法准确可靠,适用于竹叶提取物中黄酮含量的测定。竹叶提取物中7种黄酮含量在14.97~183.94 mg/g范围内,毛竹中7种黄酮总含量最高,为183.94 mg/g。不同竹种间7种黄酮成分的含量存在显著差异,荭草苷、异荭草苷和牡荆苷含量均在毛竹中最高,分别为38.45、101.30和9.42 mg/g。竹叶提取物清除DPPH自由基的IC_50_值在78.23~179.41 mg/L范围内,清除羟基自由基的IC_50_值在203.48~1250.81 mg/L范围内。毛竹叶提取物清除DPPH和羟基自由基的IC_50_值分别为78.23 mg/L和203.48 mg/L,其抗氧化活性最强,具有开发应用价值。相关性分析结果表明,荭草苷、异荭草苷含量与竹叶提取物抗氧化活性密切相关。

## Abstract (en)

The flavonoid contents of different bamboo-leaf extracts and their relationships to antioxidant activity were investigated in this study by preparing nine samples using two commercially available bamboo-leaf extract products and seven bamboo-leaf extracts such as *Phyllostachys edulis*. A high performance liquid chromatography (HPLC) method was established to determine seven flavonoid components (orientin, isoorientin, vitexin, isovitexin, tricin, luteolin and luteoloside) in these samples, which were separated using a SymmetryShield^TM^ RP8 column (250 mm×4.6 mm, 5 μm) under gradient-elution conditions using acetonitrile as mobile phase A and 0.5% (v/v) acetic acid aqueous solution as mobile phase B. The antioxidant activities of the samples were evaluated using the 1,1-diphenyl-2-picrylhydrazyl (DPPH) and hydroxyl radical-scavenging assays, with half inhibitory concentration (IC_50_) as an indicator and the butylated hydroxytoluene (BHT) and *tert*-butylhydroquinone (TBHQ) antioxidants as positive controls. Pearson correlation was then used to analyze the relationship between flavonoid content and antioxidant activity. The HPLC method was found to be accurate and reliable for determining the flavonoid contents of the bamboo-leaf extracts. The seven flavonoids were well separated, and good linear relationships were exhibited (correlation coefficients (*R*^2^)≥0.9990). Furthermore, the contents of the seven flavonoids in the bamboo-leaf extracts ranged from 14.97 to 183.94 mg/g, with the highest content of 183.94 mg/g recorded for *Phyllostachys edulis*. The bamboo species exhibited significantly different flavonoid contents, with *Phyllostachys edulis* showing the highest orientin, isoorientin, and vitexin levels of 38.45, 101.30, and 9.42 mg/g, respectively. Moreover, the bamboo-leaf extracts exhibited IC_50_ values of 78.23-179.41 mg/L for DPPH-radical-scavenging, while values of 203.48-1250.81 mg/L were recorded for hydroxyl radicals. The *Phyllostachys edulis* leaf extract exhibited the strongest antioxidant activity, with the lowest IC_50_ values of 78.23 and 203.48 mg/L for DPPH and hydroxyl, respectively; it showed greatly significant for the further development and application of *Phyllostachys edulis*. Finally, the relationships between flavonoid content and the DPPH- and hydroxyl-radical-scavenging activities (based on the IC_50_ values) were correlated, which revealed that the orientin and isoorientin contents are closely related to the antioxidant activities of the bamboo-leaf extracts. Consequently, the orientin and isoorientin contents can be used as indicators for evaluating the antioxidant activities of bamboo-leaf extracts.

中国是世界上竹资源最为丰富的国家之一,有44属857种竹类植物,约占全球竹种的50%以上,主要分布在北纬35°以南地区^[1]^,极具生态和经济价值。竹叶作为清热药材,有着悠久的药用历史^[2]^。近年来关于竹叶提取物中主要活性成分及其药理功能的开发和研究一直备受关注。黄酮类化合物作为竹叶中最具代表性的活性成分之一^[3]^,具有抗氧化^[4⇓⇓-7]^、抗菌消炎^[7,8]^、调节血脂^[9,10]^和抗细胞衰老^[11]^等作用,在医药、食品、日化产品、养殖业等方面有着广泛的应用。研究表明,竹叶中除常见的荭草苷、异荭草苷、牡荆苷和异牡荆苷4种碳苷黄酮^[12]^外,还含有苜蓿素^[13]^、木犀草素^[14]^、木犀草苷^[13]^、芹菜素^[15]^和槲皮素^[16]^等黄酮类化合物。黄酮成分含量的测定方法多采用高效液相色谱法(HPLC)^[12,17]^和高效薄层色谱法(HPTLC)^[18]^。总黄酮含量的测定多采用分光光度法,以芦丁为对照品,采用硝酸铝-亚硝酸钠比色法^[19]^进行测定。

目前关于竹叶黄酮定量及定性分析的研究涉及的竹种主要为簕竹属(*Bambusa*)、刚竹属(*Phyllostachys*)、大明竹属(*Pleioblastus*)和箬竹属(*Indocalamus*)等,仍有竹种如唐竹属(*Sinobambusa*)、矢竹属(*Pseudosasa*)、箭竹属(*Fargesia*)等较少或尚未被定量研究。市售用作抗氧化物的竹叶提取物多以淡竹叶(*Lophatherum gracile*)为原料,市场效益极大。本实验对2种市售竹叶提取物和毛竹(*Phyllostachys edulis*)等7种竹种的竹叶提取物进行黄酮成分测定及抗氧化比较,建立了同时测定竹叶提取物中7种黄酮含量的HPLC方法,并结合1,1-二苯基-2-三硝基苯肼(DPPH)和羟基自由基清除试验评价竹叶提取物的抗氧化活性差异,探讨黄酮含量与抗氧化能力的相关性,筛选优质竹叶原料,为更加合理地开发和利用竹叶黄酮资源提供参考依据。

## 1 实验部分

### 1.1 仪器、试剂与材料

2695型高效液相色谱仪配紫外检测器,美国Waters公司;MP5002、BP221S电子分析天平,德国Sartorius公司;旋转蒸发仪,日本EYELA公司;冷冻干燥机,美国Labconco公司;超纯水机,美国Pall公司;Lambda 35紫外分光光度计,美国Perkin Elmer公司;KQ-800E超声波发生仪,昆山市超声仪器有限公司;恒温水浴锅,北京市长风仪器仪表公司。

竹叶提取物商品1(标记为BLE1),竹叶黄酮含量38.82%,金华贝康生物科技开发有限公司;竹叶提取物商品2(标记为BLE2),竹叶黄酮含量39.99%,安吉圣氏生物制品有限公司;7种竹种竹叶2019年11月采于江西省林科院竹种园,经专家鉴定竹种,信息如下:唐竹属的唐竹(*S. tootsik* (Sieb.) Makino)、白皮唐竹(*S. farinosa* (McClure) Wen)、光叶唐竹(*S. tootsik* (Sieb.) Makino var. *tenuifolia* (Koidz.) S. Susuki);簕竹属的小叶琴丝竹(*B. multiplex* (Lour.) Raeusch. cv. Alphonse-Karr R. A.)和粉单竹(*B. chungii* McClure);刚竹属的毛竹(*P. edulis* (Carriere) J. Houzeau)以及大明竹属的斑苦竹(*P. maculatus* (McClure) C. D Chu et C. S. Chao)。

荭草苷、异荭草苷、牡荆苷、异牡荆苷、木犀草素和木犀草苷标准品(纯度≥98%),上海源叶生物科技有限公司;苜蓿素标准品(纯度≥98%),对照品实验耗材中心;甲醇、乙酸和乙腈(色谱纯),美国Fisher公司;DPPH,上海安普实验科技股份有限公司;抗氧化剂:2,6-二叔丁基对甲酚(BHT)购自国药集团化学试剂有限公司,叔丁基对苯二酚(TBHQ)购自上海Aladdin公司;磷酸二氢钠(NaH_2_PO_4_·2H_2_O)、磷酸氢二钠(Na_2_HPO_4_·12H_2_O),北京化工厂;邻菲啰啉(C_12_H_8_N_2_·H_2_O)、双氧水(H_2_O_2_)、硫酸亚铁(FeSO_4_·7H_2_O)、浓硫酸、无水乙醇和石油醚均为市售分析纯试剂;AB-8大孔吸附树脂,北京赛谱锐思科技有限公司。

### 1.2 竹叶提取物的制备

采用乙醇热回流提取法提取竹叶中的黄酮类化合物。将新鲜采摘的竹叶在室内环境下自然阴干,去梗后用流水式粉碎机粉碎成约40目。准确称取50 g干燥竹叶粉末,置于1000 mL圆底烧瓶中,按料液比1∶10(g/mL)加入95%乙醇水溶液500 mL,摇匀,热回流提取,温度60 ℃,回流时间6 h,操作重复3次。分别收集3次提取液合并,冷却后进行抽滤,得到粗提液。将粗提液旋转蒸发去除乙醇后,加入适量纯水补至200 mL,倒入分液漏斗中,加入石油醚600 mL进行萃取,充分振荡静置30 min,收集水相部分,萃取操作重复3次直至上层溶剂呈无色或浅色。将萃取后的水相部分减压浓缩,得到竹叶提取物浸膏。将竹叶提取物浸膏用少量40%乙醇水溶液溶解后,上样AB-8大孔吸附树脂柱,依次用纯水、20%乙醇、40%乙醇、100%乙醇洗脱,每次均为3倍柱体积,收集40%乙醇洗脱液,减压浓缩、冷冻干燥后得到竹叶提取物样品,于4 ℃冰箱保存备用。

### 1.3 溶液配制

#### 1.3.1 标准品溶液

分别精密称取荭草苷、异荭草苷、牡荆苷、异牡荆苷、苜蓿素、木犀草素和木犀草苷,加入适量甲醇,超声辅助溶解并定容于10 mL容量瓶中,得到混合标准品储备液,各成分的质量浓度分别为170、180、230、170、110、180、110 mg/L。用甲醇将储备液稀释成系列质量浓度的混合标准液,于4 ℃冰箱保存备用。将配制好的系列混合标准液过0.22 μm滤膜供HPLC检测。

#### 1.3.2 竹叶提取物样品溶液

精密称取BLE1、BLE2及1.2节制备的竹叶提取物样品各0.0200 g,加入适量甲醇,超声辅助溶解并定容于10 mL容量瓶中,得到2000 mg/L样品溶液,过0.22 μm滤膜,于4 ℃冰箱保存,供HPLC检测使用。

### 1.4 液相色谱条件

色谱柱:SymmetryShield^TM^ RP8色谱柱(250 mm×4.6 mm, 5 μm);流动相A:乙腈,流动相B: 0.5%(v/v)乙酸水溶液;柱温:30 ℃;流速:1 mL/min;检测波长:340 nm;进样量:10 μL。梯度洗脱程序:0~5 min, 15%A; 5~25 min, 15%A~18%A; 25~35 min, 18%A~30%A; 35~45 min, 30%A~50%A; 45~50 min, 50%A~60%A; 50~55 min, 60%A~15%A。

### 1.5 抗氧化活性实验

#### 1.5.1 DPPH自由基清除实验

用无水乙醇配制并稀释成300、200、110、90、70、50、40、30、20、10 mg/L的系列竹叶提取物样品溶液。设置BHT、TBHQ为阳性对照组,用无水乙醇配制并稀释成70.56、60.48、50.40、40.32、30.24、20.16、10.08 mg/L的BHT溶液和48.72、41.76、34.80、27.84、20.88、13.92、6.96 mg/L的TBHQ溶液。

参考袁凤娟等^[20]^的方法,分别取不同浓度的样品溶液1 mL,加入3 mL 51.8 mg/L的DPPH溶液于10 mL试管中混合均匀,避光条件下静置30 min,于517 nm处测定吸光值,记为*A*_1_,每组平行测定3次;用无水乙醇代替样品溶液,作为空白对照,重复上述步骤,吸光值记为*A*_0_;以无水乙醇代替DPPH溶液,重复上述步骤,测定样品本底的吸光值,吸光值记为*A*_2_。按公式(1)计算DPPH自由基清除率*Y*_1_,样品清除能力以半抑制浓度(IC_50_)值表示。


(1) *Y*_1_=[1-(*A*_1_-*A*_2_)/*A*_0_]×100%

#### 1.5.2 羟基自由基清除实验

将1.3.2节竹叶提取物样品溶液用蒸馏水逐级稀释成100~1400 mg/L的系列样品溶液。设置BHT、TBHQ为阳性对照组,用蒸馏水稀释成280、220、165、110、55 mg/L的BHT溶液和402、287、230、138、46 mg/L的TBHQ溶液。

采用Fenton反应测定竹叶提取物对羟基自由基的清除实验,参考Zhang等^[21]^的方法,向10 mL试管中加入2 mL pH=7.4的0.15 mol/L磷酸盐缓冲液、1 mL 0.75 mmol/L的邻菲啰啉溶液和1 mL 0.75 mmol/L的硫酸亚铁溶液,摇匀,加入不同浓度样品溶液1 mL,摇匀,加入0.01%双氧水溶液1 mL,摇匀,于37 ℃水浴中充分反应1 h,于510 nm处测定吸光值,记为*A*_z_,每组平行测定3次。另外再做未损伤管和损伤管,重复上述步骤,其中未损伤管以等体积蒸馏水分别代替样品溶液和双氧水,吸光值记为*A*_x_;损伤管以等体积蒸馏水代替样品溶液,吸光值记为*A*_y_。同时测定样品本底的吸光值,向10 mL试管中加入2 mL磷酸盐缓冲液、3 mL蒸馏水和1 mL样品溶液,重复上述步骤,吸光值记为*A_z_'*。按公式(2)计算羟基自由基清除率*Y*_2_,样品清除能力以IC_50_值表示。


(2) Y2=Az-Az'-AyAx-Ay×100%

#### 1.5.3 数据处理

采用Microsoft excel软件对数据进行处理,分析计算清除率,IBM SPSS Statistics 25软件对清除率进行回归概率分析。

## 2 结果与讨论

### 2.1 毛竹叶提取物样品的色谱分析

按1.4节色谱条件进行检测,混合标准液与毛竹叶提取物样品的色谱图见图1, 7种黄酮成分的色谱峰峰形窄且对称,分离效果较好。将竹叶提取物样品的色谱图与标准品色谱图进行对比,结合相应成分的保留时间,可对竹叶提取物中7种黄酮成分进行定性分析。

**图1:**
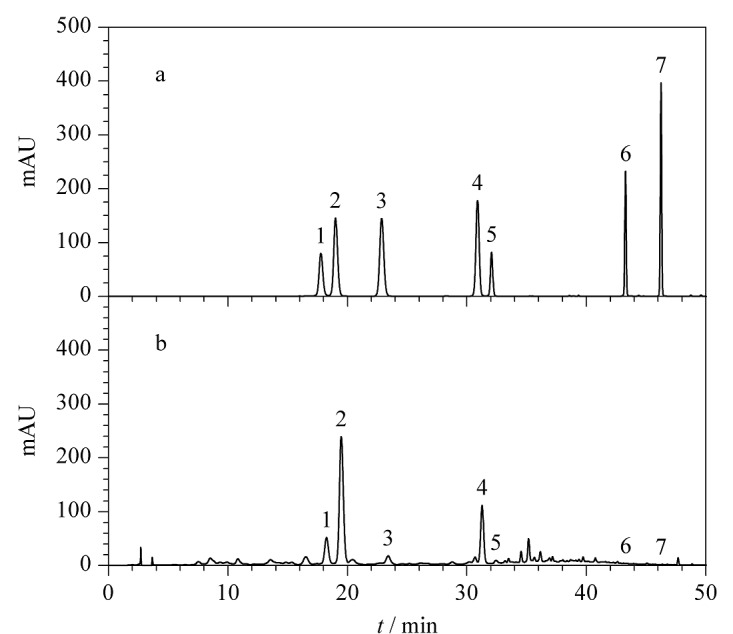
(a)混合标准液和(b)毛竹叶提取物样品的色谱图

### 2.2 方法学考察

#### 2.2.1 线性关系、检出限和定量限

将1.3.1节系列混合标准液按1.4节色谱条件进行检测,以色谱峰的峰面积(*y*)对质量浓度(*x*)进行线性回归,得到各个黄酮成分的线性回归方程、线性范围和相关系数(*R*^2^)。用甲醇逐级稀释混合标准液并进行HPLC测定,当信噪比(*S/N*)=3时为检出限(LOD), *S/N*=10时为定量限(LOQ),测定结果见表1。各成分峰面积与质量浓度在各自范围内线性关系良好(*R*^2^≥0.9990),检出限在0.20~0.48 mg/L范围内,定量限在0.50~1.20 mg/L范围内,表明仪器灵敏度较高,能够实现对样品中较低浓度成分的检测。

**表1:** 7种黄酮成分的线性关系、相关系数、检出限和定量限

Component	Linear equation	Linear range/(mg/L)	R^2^	LOD/(mg/L)	LOQ/(mg/L)
Orientin	y=15836x-51328	1.13-170	0.9993	0.40	0.90
Isoorientin	y=26962x-115503	1.20-180	0.9990	0.48	1.20
Vitexin	y=21948x+8583.1	1.53-230	0.9996	0.40	1.10
Isovitexin	y=30338x-23490	1.13-170	0.9999	0.25	0.80
Luteoloside	y=15355x+18309	1.00-110	0.9992	0.40	0.80
Tricin	y=24059x+19988	0.73-110	0.9993	0.21	0.70
Luteolin	y=26111x-70153	1.20-120	0.9990	0.20	0.50

*y*: peak area; *x*: mass concentration, mg/L.

#### 2.2.2 仪器和方法精密度

取任意一混合标准液按1.4节色谱条件连续进样5次,测定各成分峰面积并计算相应的相对标准偏差(RSD),考察仪器精密度。实验结果显示,7种黄酮成分峰面积的RSD值均在2%以内,表明仪器精密度良好。

按1.3.2节平行配制毛竹叶提取物样品6份,按1.4节色谱条件进行检测,测定各成分峰面积,计算各成分的含量及相应的RSD,考察方法重复性。实验结果显示,毛竹叶提取物样品中7种黄酮成分含量的RSD值均在5%以内,表明该方法重复性良好。

#### 2.2.3 稳定性

取任意一混合标准液按1.4节色谱条件,在1天内分别于0、3、6、9、12 h进行检测,测定各成分峰面积并计算日内RSD;在3天内分别于同一时间进行检测,测定各成分峰面积并计算日间RSD,评估日内和日间稳定性。结果显示,7种黄酮成分的峰面积的日内和日间RSD均在4%以内,表明这7种黄酮成分在12 h和3天内稳定性良好。

#### 2.2.4 加标回收率

精密称取3份毛竹叶提取物样品0.0020 g,分别添加低、中、高3个水平的混合标准液1 mL,超声辅助溶解,过0.22 μm滤膜,按1.4节色谱条件进行检测,测定各成分峰面积,计算加标回收率和相应RSD。7种黄酮成分加标回收率的测定结果见表2。7种黄酮成分的加标回收率在92.96%~109.33%范围内,RSD均在4.06%以内,表明该测定方法准确性良好,适用于竹叶提取物中黄酮成分的含量测定。

**表2:** 7种黄酮成分的加标回收率(*n*=3)

Component	Original/ (mg/g)	Added/ (mg/g)	Measured/ (mg/g)	Recovery/ %	RSD/ %
Orientin	38.55	17.00	54.36	92.96	4.06
		26.98	64.70	96.74	1.66
		53.97	91.20	97.57	1.28
Isoorientin	94.80	18.00	112.10	95.90	3.91
		28.57	122.93	98.43	1.04
		57.14	151.42	99.08	0.49
Vitexin	8.94	23.00	31.38	97.57	1.56
		36.51	45.42	99.94	0.50
		73.02	82.08	100.18	0.81
Isovitexin	30.11	17.00	46.97	99.18	2.55
		26.98	55.43	93.84	2.35
		53.97	82.50	97.09	0.41
Luteoloside	2.92	11.00	13.86	99.42	2.05
		17.46	20.43	100.29	1.60
		34.92	36.98	97.53	0.92
Tricin	0.11	11.00	11.10	99.92	1.21
		17.46	18.66	106.25	1.00
		34.92	35.51	101.37	0.86
Luteolin	0.17	18.00	19.68	108.35	1.55
		28.57	30.13	104.84	1.40
		57.14	62.65	109.33	1.24

### 2.3 竹叶提取物中7种黄酮成分的含量分析

按1.4节色谱条件对1.3.2节竹叶提取物样品进行检测,每个样品重复测定3次,以标准品与竹叶提取物的保留时间和紫外光谱图对样品中的各成分进行定性,测定各成分的峰面积并计算相应的含量,IBM SPSS Statistics 25软件对数据进行单因素方差分析及邓肯式新复极差检验。9种竹叶提取物样品中7种黄酮成分的含量及差异性结果见表3。由表3可知,样品中7种黄酮总含量在14.97~183.94 mg/g范围内,其中毛竹的总含量最高,为183.94 mg/g,明显高于其他竹种;而白皮唐竹和光叶唐竹的总含量相对较低,分别为14.97 mg/g和16.36 mg/g。竹种间7种黄酮成分的含量存在较大差异,荭草苷、异荭草苷和牡荆苷含量均在毛竹中最高,分别为38.45、101.30和9.42 mg/g,毛竹中异荭草苷含量显著高于毛竹中其他黄酮成分含量,且显著高于其他竹种中的异荭草苷含量;异牡荆苷含量在小叶琴丝竹中最高,为42.56 mg/g,但小叶琴丝竹中未检测到荭草苷成分;木犀草苷含量在粉单竹和斑苦竹中相对较高。唐竹属除唐竹检出少量的木犀草素外均未检出苜蓿素和木犀草素,其他属竹种所含苜蓿素和木犀草素含量均比较低。2种市售竹叶提取物中均含有较高含量的荭草苷和异荭草苷,而毛竹叶提取物中荭草苷和异荭草苷含量显著高于2种市售竹叶提取物。

**表3:** 9种竹叶提取物中7种黄酮成分的含量(*n*=3)

Component	BLE1	BLE2	S. tootsik	S. farinosa	S. tenuifolia	B. multiplex	B. chungii	P. edulis	P. maculatus
Orientin	16.74±0.12^c^	17.46±0.16^d^	4.31±0.10^b^	2.23±0.05^a^	2.46±0.11^a^	-	1.88±0.02^a^	38.45±1.17^e^	2.27±0.04^a^
Isoorientin	34.59±0.41^e^	41.14±0.73^f^	6.78±0.07^b^	4.61±0.08^ab^	5.12±0.06^ab^	29.85±1.00^d^	13.23±0.27^c^	101.30±4.82^g^	2.71±0.02^a^
Vitexin	4.98±0.16^f^	4.37±0.17^e^	0.67±0.02^b^	1.78±0.06^d^	4.30±0.28^e^	0.99±0.08^c^	0.29±0.03^a^	9.42±0.37^g^	4.23±0.09^e^
Isovitexin	11.50±0.04^c^	15.44±0.14^d^	1.76±0.04^a^	1.00±0.06^a^	1.27±0.07^a^	42.56±1.74^g^	19.24±1.01^e^	31.14±1.26^f^	4.53±0.09^b^
Luteoloside	0.36±0.01^a^	0.29±0.01^a^	8.11±0.17^d^	5.35±0.04^c^	3.21±0.23^b^	15.39±0.26^e^	16.76±1.76^f^	3.36±0.11^b^	17.68±0.26^f^
Tricin	0.056±0.001^a^	0.052±0.001^a^	-	-	-	0.035±0.008^a^	0.831±0.018^d^	0.103±0.005^b^	0.359±0.035^c^
Luteolin	0.134±0.003^b^	0.790±0.017^g^	0.104±0.005^a^	-	-	0.363±0.020^f^	0.332±0.016^e^	0.162±0.006^c^	0.189±0.013^d^
Total	68.36	79.54	21.73	14.97	16.36	89.19	52.56	183.94	31.97

BLE: commercially available bamboo-leaf extract product; -: not detected. The different letters in the same row indicate that there are significant differences at 0.05 level (mean±standard deviation, *n*=3).

### 2.4 竹叶提取物的抗氧化活性分析

以样品对DPPH和羟基自由基的IC_50_值作评价指标,IC_50_值越小表明样品清除自由基能力越强。以抗氧化剂BHT和TBHQ为阳性对照,比较竹叶提取物抗氧化活性的强弱。样品对DPPH和羟基自由基清除率IC_50_值见表4。

**表4:** 样品对DPPH和羟基自由基的清除能力

Sample	IC_50_/(mg/L)	
	DPPH·	·OH
BHT	62.75	156.72
TBHQ	19.68	38.73
BLE1	97.24	379.05
BLE2	93.49	239.25
S. tootsik	150.29	601.22
S. farinosa	179.41	1232.54
S. tenuifolia	146.03	1250.81
B. multiplex	155.87	685.57
B. chungii	96.41	273.38
P. edulis	78.23	203.48
P. maculatus	157.67	508.41

IC_50_: half inhibitory concentration; DPPH: 1,1-diphenyl-2-picrylhydrazyl; BHT: butylated hydroxytoluene; TBHQ: *tert*-butylhydroquinone.

#### 2.4.1 DPPH自由基清除能力

由表4可知,样品对DPPH自由基的IC_50_值在19.68~179.41 mg/L范围内,样品对DPPH自由基清除能力由强到弱依次为TBHQ>BHT>毛竹>BLE2>粉单竹>BLE1>光叶唐竹>唐竹>小叶琴丝竹>斑苦竹>白皮唐竹。竹叶提取物样品均具有较好的DPPH自由基清除能力,其中毛竹、粉单竹竹叶提取物IC_50_值为78.23、96.41 mg/L,是实验制备的竹叶提取物样品中最低的,清除效果最好。抗氧化剂BHT和TBHQ的IC_50_值分别为62.75、19.68 mg/L,毛竹、粉单竹竹叶提取物IC_50_值接近抗氧化剂BHT的IC_50_值,表明毛竹、粉单竹竹叶提取物具有和抗氧化剂BHT基本相当的DPPH自由基清除能力。BLE1和BLE2的IC_50_值分别为97.24和93.49 mg/L,表明市售竹叶提取物具有较好的DPPH自由基清除能力,毛竹叶提取物的DPPH自由基清除能力还要优于BLE1和BLE2。

#### 2.4.2 羟基自由基清除能力

由表4可知,样品对羟基自由基的IC_50_值在38.73~1250.81 mg/L范围内,样品对羟基自由基清除能力由强到弱依次为TBHQ>BHT>毛竹>BLE2>粉单竹>BLE1>斑苦竹>唐竹>小叶琴丝竹>白皮唐竹>光叶唐竹,竹叶提取物样品均具有较好的羟基自由基清除能力,其中毛竹、粉单竹竹叶提取物IC_50_值为203.48、273.38 mg/L,是实验制备的竹叶提取物样品中最低的,清除效果最好。白皮唐竹和光叶唐竹竹叶提取物对羟基自由基的IC_50_值相对偏高,为1232.54、1250.81 mg/L,对羟基自由基的清除效果不如其他实验竹种竹叶。抗氧化剂BHT和TBHQ的IC_50_值分别为156.72、38.73 mg/L,毛竹、粉单竹竹叶提取物IC_50_值接近抗氧化剂BHT的IC_50_值,表明毛竹、粉单竹竹叶提取物具有和抗氧化剂BHT基本相当的羟基自由基清除能力。BLE1和BLE2的IC_50_值分别为379.05、239.25 mg/L,表明市售竹叶提取物具有较好的羟基自由基清除能力,毛竹竹叶提取物具有优于BLE1、BLE2,以及粉单竹竹叶提取物具有优于BLE1的羟基自由基清除能力。

综上所述,毛竹和粉单竹竹叶提取物均表现出较好的DPPH和羟基自由基清除能力,具有和抗氧化剂BHT基本相当的抗氧化活性,毛竹叶提取物具有优于市售用作抗氧化剂的2种竹叶提取物商品的抗氧化活性,由于毛竹叶提取物是天然产物,绿色健康,且资源丰富,因此,毛竹叶提取物可以用作天然抗氧化剂,极具开发和应用价值。

### 2.5 黄酮含量与IC_50_值相关性分析

采用IBM SPSS Statistics 25软件对9种竹叶提取物样品中各黄酮含量与清除DPPH和羟基自由基能力的IC_50_值进行Pearson相关性分析,相关性系数及显著性结果见表5。由表5可知,除木犀草苷与竹叶提取物对DPPH的IC_50_值显示正相关,与竹叶提取物对羟基自由基的IC_50_值存在较弱的负相关外,其他6种黄酮均与竹叶提取物对DPPH和羟基自由基的IC_50_值存在一定的负相关性。竹叶提取物对DPPH的IC_50_值与荭草苷、异荭草苷含量呈显著负相关(*P*<0.05),相关系数分别为-0.752、-0.717;其次与牡荆苷呈较强负相关,相关系数为-0.537。竹叶提取物对羟基自由基的IC_50_值与荭草苷、异荭草苷和木犀草素含量呈较强负相关,相关系数分别为-0.562、-0.572和-0.596。综上所述,说明荭草苷、异荭草苷含量与竹叶提取物的抗氧化活性密切相关,荭草苷、异荭草苷含量可以作为评价竹叶提取物抗氧化活性的指标。

**表5:** 竹叶提取物中7种黄酮含量与清除DPPH和羟基自由基的IC_50_值的相关性分析

Indicator	DPPH·	·OH
Orientin	-0.752^*^	-0.562
Isoorientin	-0.717^*^	-0.572
Vitexin	-0.537	-0.302
Isovitexin	-0.384	-0.462
Luteoloside	0.354	-0.071
Tricin	-0.293	-0.418
Luteolin	-0.453	-0.596

* Significant correlation (*P*<0.05).

## 3 结论

本实验建立了同时测定竹叶提取物中荭草苷、异荭草苷、牡荆苷、异牡荆苷、木犀草苷、苜蓿素和木犀草素7种黄酮含量的HPLC方法。毛竹叶提取物在荭草苷、异荭草苷含量及对DPPH、羟基自由基清除效果方面均优于2种市售竹叶提取物,且具有与抗氧化剂BHT基本相当的抗氧化活性,是良好的天然抗氧化物,绿色健康且资源丰富,极具开发和应用价值。相关性分析结果表明,荭草苷、异荭草苷含量与竹叶提取物抗氧化活性密切相关,因此荭草苷、异荭草苷可以作为评价竹叶提取物抗氧化能力的指标。此外,关于竹叶提取物中黄酮成分与抗氧化活性的关系,究竟是单一黄酮成分起作用,还是多种黄酮成分协同作用,抑或是多种黄酮成分间存在着协同、拮抗、竞争等作用,亟待进一步研究。
